# Acanthosis nigricans – a potentially useful clue to the presence of significant occult disease at autopsy

**DOI:** 10.1007/s12024-024-00815-6

**Published:** 2024-04-11

**Authors:** Roger W. Byard, John Gilbert

**Affiliations:** 1 Forensic Science SA, Adelaide, South Australia 5000 Australia; 2 School of Biomedicine, The University of Adelaide, Level 2, Room N237, Helen Mayo North, Frome Road, Adelaide, SA 5005 Australia

**Keywords:** Acanthosis nigricans, Diabetes mellitus, Morbid obesity, Insulin resistance, Occult disease, Autopsy, Cause of death

## Abstract

A 19-year-old male was found dead in his apartment. At autopsy he was morbidly obese (Body mass index; BMI – 40.5) with multiple areas of velvety pigmented thickening of the skin in folds around the neck, in the axillae, in the inframammary regions, over the anterior waistline and groin regions and over the dorsal aspects of the feet. These had the typical appearance of acanthosis nigricans. Internal examination revealed aspiration of gastric contents into the airways. Vitreous humour biochemistry showed markedly elevated levels of both glucose (62.9 mmol/L) and β-hydroxybutyrate (13.54 mmol/L). Death was, therefore, due to aspiration pneumonia complicating diabetic ketoacidosis on a background of morbid obesity. The initial indicator of underlying diabetes, in conjunction with obesity had been acanthosis nigricans.

## Case report

A 19-year-old socially-isolated Chinese student was found dead in his apartment. He had complained to his family that he was experiencing weakness, fevers, muscle aches and cramping in the weeks prior to death and had been vomiting but had not sought medical attention. There was no significant past history, in particular no history of diabetes mellitus.

**Fig. 1:**
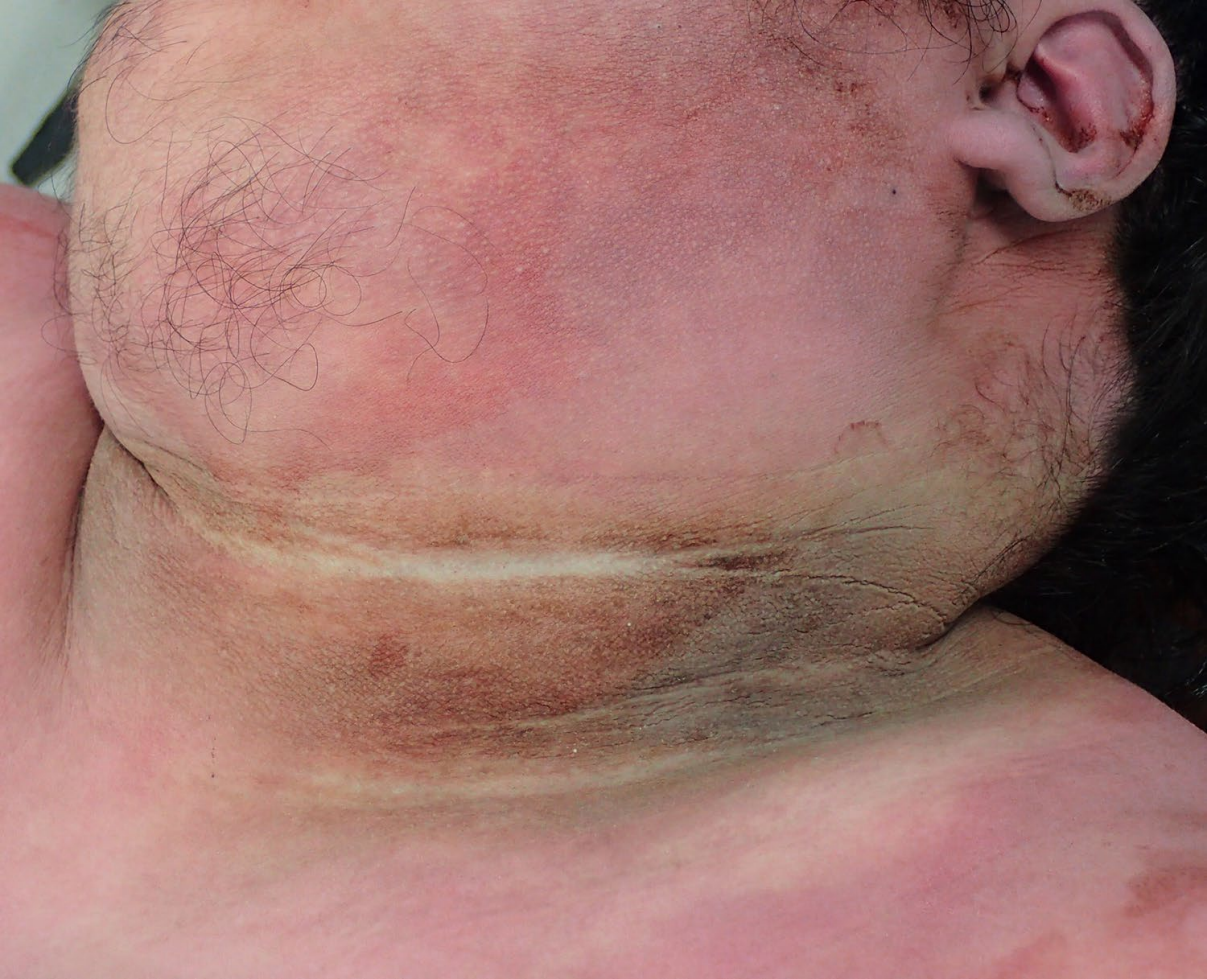
Characteristic velvety hyperpigmentation of the skin around the neck in a 19-year-old morbidly-obese male with occult diabetes mellitus

**Fig. 2:**
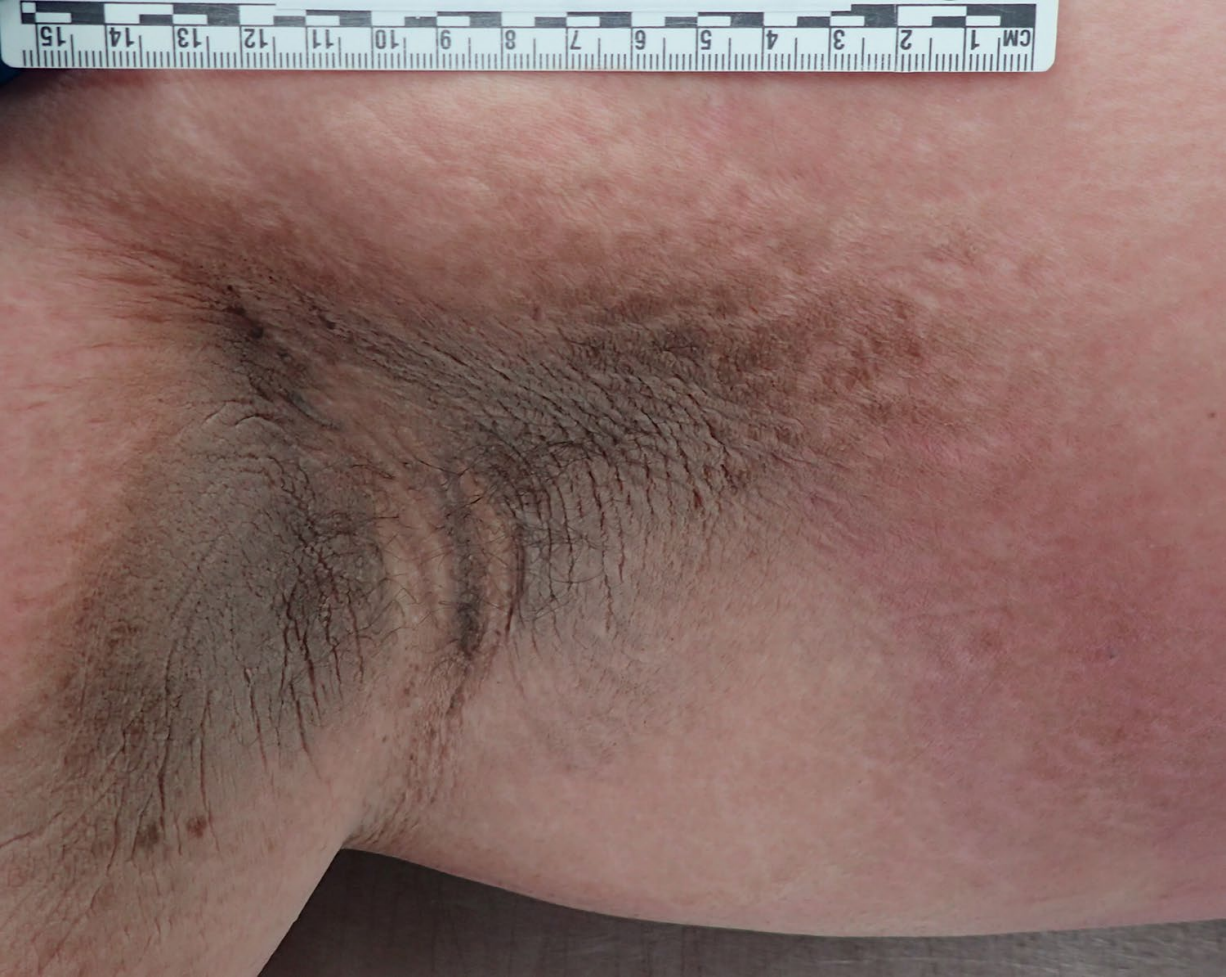
Typical hyperpigmentation of the right axilla in the same case

**Fig. 3:**
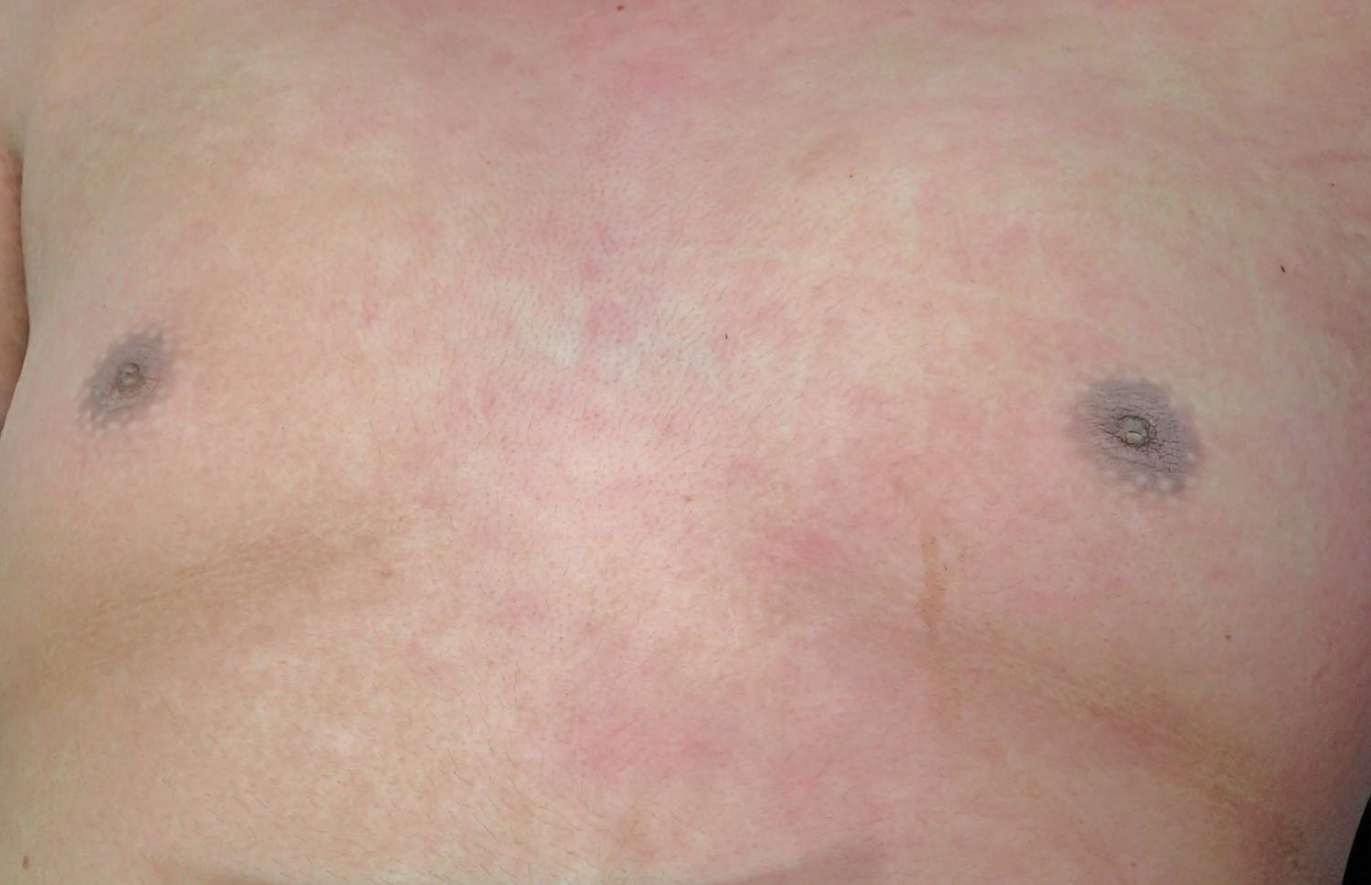
More subtle areas of bilateral pigmentation in the submammary regions in the reported case

**Fig. 4:**
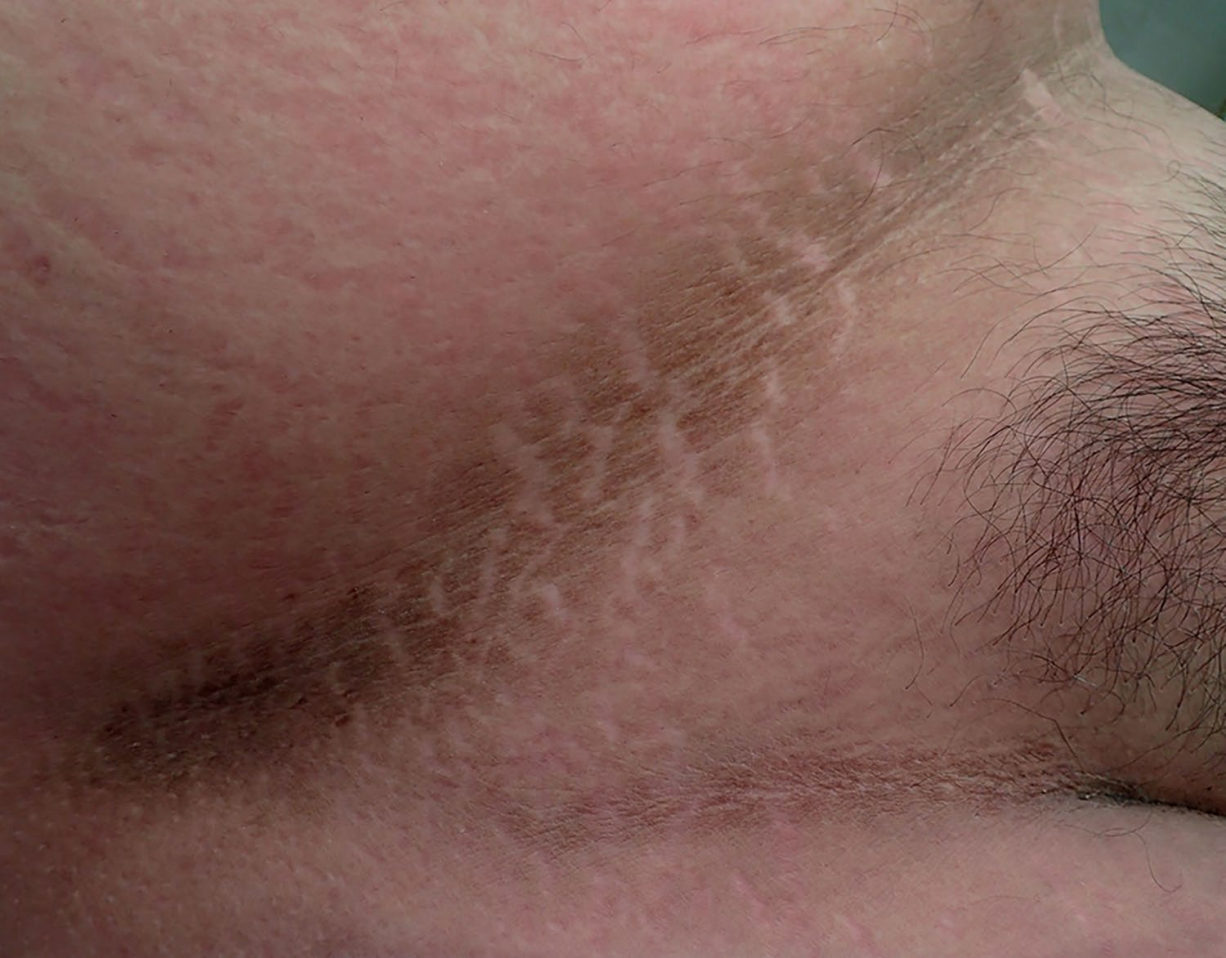
A line of pigmentation representing acanthosis nigricans in a fat fold on the lower anterior abdominal wall in the same case

At autopsy the height was 175 cm and the weight was 124 kg (Body mass index; BMI – 40.5 – morbidly obese). Abdominal striae were present in addition to velvety pigmented thickening of the skin in skin folds around the neck (Fig. 1), in the axillae (Fig. 2), in the inframammary regions (Fig. 3), over the anterior waistline (Fig. 4) and groin regions and over the dorsal aspects of the feet. This had the typical appearance of acanthosis nigricans.

Internal examination revealed congestion and edema of the lungs with aspiration of gastric contents, steatosis of the liver and cortical pallor of the kidneys. Dipstick testing of the urine showed high levels of glucose and ketones consistent with diabetic ketoacidosis. This was confirmed by vitreous humour biochemistry with markedly elevated levels of glucose (62.9 mmol/L) and β-hydroxybutyrate (13.54 mmol/L).

**Fig. 5:**
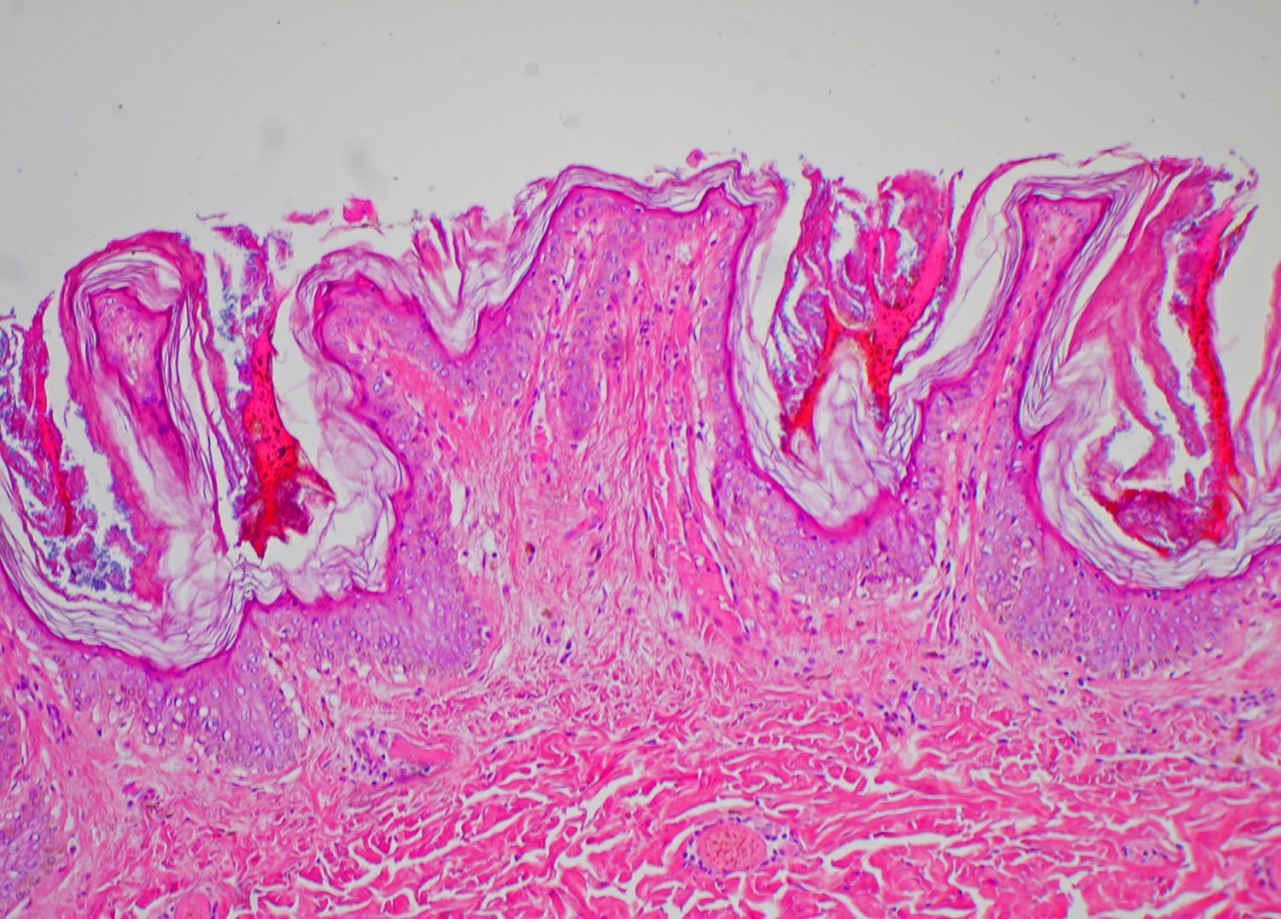
Typical papillomatosis with hyperkeratosis and papillomatosis with minimal inflammation in a section from one of the areas of acanthosis nigricans. (Hematoxylin and eosin × 60)

The kidneys showed marked autolysis which precluded histological evaluation of Armanni-Ebstein changes from hyperglycemia [1], however basal vacuolation of tubular epithelial cells, occasionally highlighted by deposition of formalin pigment, was in keeping with ketoacidosis [2]. The skin showed uniform orthokeratotic hyperkeratosis and papillomatosis consistent with acanthosis nigricans with no appreciable melanin hyperpigmentation (Fig. 5). There was widespread aspiration pneumonia in the lungs and marked steatosis of the liver. Toxicology was negative except for acetone from the ketoacidosis and there was no evidence of trauma or other underlying organic diseases that could have caused of contributed to death.

The fatal episode was, therefore, due to aspiration pneumonia complicating diabetic ketoacidosis on a background of morbid obesity. The initial indicator of underlying diabetes, in conjunction with obesity had been acanthosis nigricans.

## Discussion

Acanthosis nigricans refers to a skin condition that is characterised by irregularly-defined symmetric areas of velvety hyperpigmentation that are located primarily in skin folds such as the axillae, groins and back of the neck. Less commonly it may be found over the knees and elbows, the knuckles, scalp and around the umbilicus. It may, however, involve any area of the skin and in cases associated with malignancy can be found on mucosal surfaces such as the lips and oral cavity. In obese hyperandrogenic women without diabetes it is most often vulval. It has been described in dogs, in particular dachshunds [3]. It most often occurs in those under 40 years of age, with no sex predilection, in association with obesity, insulin-resistant diabetes mellitus, acromegaly, hypothyroidism, Addison disease, Cushing disease, and polycystic ovarian disease [4].

The first case was reported by Pollitzer in 1890 [3, 5, 6]. The development of these lesions is thought to relate to the effects of increased levels of various growth factors such as insulin-like growth factor (ILGF) and transforming growth factor (TGF) on fibroblast growth factor receptor (FGFR) and epidermal growth factor receptor (EGFR) promoting keratinocyte and fibroblast proliferation producing the typical macro and microscopic appearances [7]. The exact trigger for the development of these skin lesions is not, however, known. On microscopy the major features are papillomatosis and hyperkeratosis with minimal hyperpigmentation. Papillomatosis occurs when dermal papillae project upwards into the epidermis which may be thinned [3]. There is usually no significant inflammation unless there is a superimposed infection associated with diabetes mellitus or intertriginous chafing [8]. An increase in melanin pigmentation may occur. Papillomatosis without hyperpigmentation is more common in mucosal lesions and skin lesions may be associated with hair loss in the axillae, and in the scalp, limbs and eyebrows. There may be hyperkeratosis of the soles and palms (tylosis), particularly in cases associated with malignancy, in addition to multiple seborrheic keratoses (the sign of Leser-Trélat), and florid papillomatosis [3].

Classifications have varied from those with several subcategories such as benign, malignant and syndromic, to more detailed subdivisions of 1) benign, 2) obesity-associated, 3) syndromic, 4) malignant, 5) acral, 6) unilateral, 7) drug-induced and 8) mixed when combinations are present [3, 6].

Benign acanthosis nigricans is a rare condition which may be unilateral or generalised, present in the very young, heritable and associated with melanocytic nevi. It stops developing or decreases around puberty and is not associated with obesity or malignancy [3].

There is a well-recognised association between acanthosis nigricans and obesity with it being found in a third of obese individuals who are 120–170% above their ideal body weight, increasing to 100% in those who are more than 250% above [9]. Given that there has been a significant increase in obesity and morbid obesity in both the community and in individuals presenting to medicolegal facilities for autopsy, forensic pathologists will be seeing more of these cases in the future [10, 11]. Acanthosis nigricans is thought to develop in obese individuals due to underlying metabolic disruptions resulting in high levels of circulating insulin causing insulin-like growth factor to stimulate proliferation of keratinocytes and dermal fibroblasts [12]. It is important, however, not to merely attribute acanthosis nigricans to obesity without consideration of a number of quite significant other associated conditions as it is may be a very distinct cutaneous marker for certain systemic diseases.

A wide variety of syndromes may give rise to acanthosis nigricans ranging from leprechaunism to Hashimoto thyroiditis and polycystic ovary disease. A comprehensive list of associated syndromes has been provided by Schwartz, including possible differential diagnoses [3]. One of the most important clinical associations, as was demonstrated in the reported case, is diabetes mellitus with a prevalence ranging from 19.4 to as high as of 71% in some studies [7, 13, 14]. It has been suggested that acanthosis nigricans in the living may be used as a marker for high risk patients [15], particularly if it is also associated with obesity in childhood and adolescence [16–19].

Malignant or paraneoplastic acanthosis nigricans may be a clue to an underlying tumor such as gastric adenocarcinoma. While acanthosis nigricans may develop concomitantly with other symptoms and signs of malignancy it may also be the first indication of disease. Other malignancies of the bladder, kidney, ovary, pancreas, prostate, esophagus, intestines, thyroid gland, uterus and bile ducts may also manifest in this way. It has been reported in children with Wilms tumor [3, 6, 20]. There may also be a history of quite rapid development of cutaneous lesions in cases with underlying cancers.

Acral acanthosis nigricans consists of thickening of the dorsal skin of the hands and feet that may be observed in dark-skinned individuals. It is usually benign although a case with metastatic dermatofibrosarcoma has been reported. Unilateral acanthosis nigricans has been regarded as a nevoid disorder which may be inherited. It is not associated with an endocrine disorder, syndrome or malignancy [3].

Drug-induced acanthosis nigricans may be associated with an array of prescribed medications that may induce hyperinsulinemia such as steroids, oral contraceptives, estrogen, insulin and nicotinic acid [21]. Thus, a history of prescribed drugs may provide useful information at autopsy to explain the typical skin lesions. The lesions should usually have resolved if the medication had been ceased [6]. Finally, certain individuals may exhibit more than one type.

In conclusion, the reported case has demonstrated previously unsuspected diabetic ketoacidosis in a morbidly obese individual heralded by the presence of striking acanthosis nigricans. Although commonly associated with obesity, acanthosis nigricans may be a valuable clue for the presence of a wide array of other syndromic, metabolic and malignant conditions. Thus, if acanthosis nigricans is observed during an external examination, serious consideration should be given to performing a full autopsy with internal evaluation of body cavities with histology and biochemical testing (Table 1).

**Table 1:** Conditions to consider when acanthosis nigricans is identified at autopsy

**Benign**
**Obesity-associated**
**Syndromic/endocrine**
Diabetes mellitus
**Malignant**
Gastric carcinoma
**Acral**
**Unilateral**
**Drug-Induced**
**Mixed**

## Data Availability

There is no original data other than the case details.
